# MetWAMer: eukaryotic translation initiation site prediction

**DOI:** 10.1186/1471-2105-9-381

**Published:** 2008-09-18

**Authors:** Michael E Sparks, Volker Brendel

**Affiliations:** 1 Department of Genetics, Development and Cell Biology, Iowa State University, Ames, IA 50011, USA; 2 Department of Statistics, Iowa State University, Ames, IA 50011, USA

## Abstract

**Background:**

Translation initiation site (TIS) identification is an important aspect of the gene annotation process, requisite for the accurate delineation of protein sequences from transcript data. We have developed the MetWAMer package for TIS prediction in eukaryotic open reading frames of non-viral origin. MetWAMer can be used as a stand-alone, third-party tool for post-processing gene structure annotations generated by external computational programs and/or pipelines, or directly integrated into gene structure prediction software implementations.

**Results:**

MetWAMer currently implements five distinct methods for TIS prediction, the most accurate of which is a routine that combines weighted, signal-based translation initiation site scores and the contrast in coding potential of sequences flanking TISs using a perceptron. Also, our program implements clustering capabilities through use of the *k*-medoids algorithm, thereby enabling cluster-specific TIS parameter utilization. In practice, our static weight array matrix-based indexing method for parameter set lookup can be used with good results in data sets exhibiting moderate levels of 5'-complete coverage.

**Conclusion:**

We demonstrate that improvements in statistically-based models for TIS prediction can be achieved by taking the class of each potential start-methionine into account pending certain testing conditions, and that our perceptron-based model is suitable for the TIS identification task. MetWAMer represents a well-documented, extensible, and freely available software system that can be readily re-trained for differing target applications and/or extended with existing and novel TIS prediction methods, to support further research efforts in this area.

## Background

Translation initiation in eukaryotic mRNA molecules typically follows the basic mechanism postulated by the scanning hypothesis [1], according to which the 40S ribosomal subunit binds to the 5'-cap of an mRNA, scans in the 5' → 3' direction until the first AUG is encountered, stalls to recruit the 60S subunit, and forms the 80S ribosomal particle, which then proceeds unencumbered with translation to render a protein product (reviewed in [2]). Roughly 10% of eukaryotic transcripts are subject to so-called leaky scanning [3], in which the ribosome continues scanning beyond the first AUG codon until it encounters one in a more favorable context [4]. Alternative methods to initiate translation from certain RNAs of viral origin exist, including, one, the formation of kissing stem-loops to facilitate translation initiation from a 5'-proximal methionine codon [5] and, two, usage of internal ribosomal entry sites [6]. Efficient translation initiation from non-methionine codons is also possible in eukaryotes [7,8]. In the present work, we are concerned only with modeling 5'-cap-dependent translation initiation occurring at AUG codons in eukaryotic protein coding genes of non-viral origin.

A variety of approaches to *in silico *translation initiation site (TIS) detection in nucleotide sequences have been previously considered, including perceptrons [9], single, multilayer artificial neural networks (ANNs) [10], multiple, multilayer ANNs [11], linear discriminant analysis [12], mixture Gaussian models [13], unsupervised clustering algorithms [14], support vector machines [15-17], expectation maximization [18], and hidden Markov models [19]. Unfortunately, none of these methods are conveniently available in the form of open source, distributed software. In part, our motivation for this work is to provide a software framework for the implementation and testing of a variety of different algorithmic approaches to TIS identification. Software systems such as ESTScan [20,21] and Diogenes [22], originally developed for detecting significant open reading frames in (potentially errant) cDNA sequences, have also been used to identify TISs, although empirical results suggest that these methods are inappropriate for the task [23]. One strategy for integrating TIS detection methods into computational gene finding pipelines, as opposed to predicting TISs in mRNA sequences *per se*, is to refine results produced from a separate gene finding tool. For example, the TICO tool [14,24] was developed to refine prokaryotic gene structure annotations generated by the GLIMMER program [25,26]. The mechanism of translation initiation in prokaryotes differs considerably from that of eukaryotes [27]. Here, we describe the MetWAMer system, developed primarily for post-processing spliced alignment-based eukaryotic gene annotation results provided in the gthXML format [28]. A variant of the MetWAMer code is abstracted from any specific gene prediction system and allows TIS prediction in eukaryotic reading frames as generated by any procedure, thus facilitating integration into other gene prediction software and workflows.

In the following we first describe MetWAMer and its incorporated TIS-finding algorithms and then discuss applications to annotating transcripts from the model plant *Arabidopsis thaliana*. MetWAMer currently implements five distinct methods for TIS detection. Among these, the best performer is the perceptron-based flank-contrasting weighted log-likelihood ratio routine (PFCWLLKR), which combines local TIS feature scores and scores probing the contrast in coding potential of sequences flanking a site. MetWAMer allows the user to develop and apply stratified parameter sets for an arbitrary number of data clusters. We demonstrate the potential for stratified parameter deployment to yield considerable increases in TIS prediction accuracy relative to a homogeneous parameter strategy. Also discussed are strategies for parameter selection in practice, depending on prior assessment of the likelihood that the transcript under consideration is or is not 5'-complete. Source code implementing this package is released under the ISC license, and is available for download from [29]. It is also registered as Additional File 1 in this report.

## Implementation

In the following subsection, we briefly describe the components of the MetWAMer software. Then we discuss the distinct algorithms implemented for TIS-identification and report our training and testing approaches for *Arabidopsis *data.

### The *MetWAMer *system

The MetWAMer code, written in the C programming language, implements the executable files MetWAMer.CDS and MetWAMer.gthXML. MetWAMer.CDS is the generic application for TIS prediction in eukaryotic open reading frames, as derived via any computational procedure. MetWAMer.gthXML is a special-purpose variant of the software, specifically tailored to refine gene structure predictions generated by the GenomeThreader [30] and GeneSeqer [31] programs for spliced alignment-based gene structure annotation. GenomeThreader and GeneSeqer, like most other spliced-alignment tools, do not make explicit predictions concerning translation initiation sites, but rather are restricted to the identification of reading frames in genomic sequences for which transcript evidence or homologous sequences suggest a protein coding function. MetWAMer.gthXML extends the 5'- and 3'-most termini of these annotated reading frames such that a maximal (non-stop) open reading frame (ORF) is realized. (No distinction between MetWAMer.gthXML and the more generic MetWAMer.CDS variant exists subsequent to reading frame maximization; we therefore refer to the system as "MetWAMer" for the remainder of this article.) MetWAMer scans for methionine-encoding sites in this maximal reading frame, considering their potential as translation initiation sites under a variety of scoring schemes, described below, in an attempt to identify a TIS for the gene structure under consideration. At most one prediction per maximal ORF is made, if and only if the optimal solution rendered exceeds some method-specific quality threshold.

**Figure 1:**
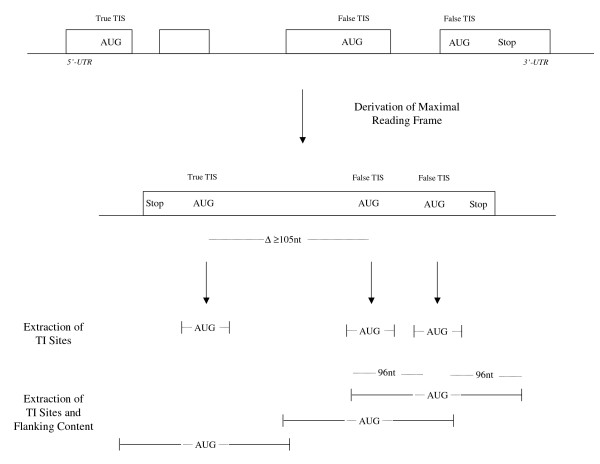
**Extraction of training data**. A genomic protein coding sequence is conceptually spliced into an open reading frame, which is extended at its 5'- and 3'-termini to render a maximal (non-stop) reading frame. For LLKR, WLLKR, and BAYES, only sequences comprising the immediate context of true and false TISs (defined as five bases upstream through three bases downstream of the ATG codon's adenine residue) are extracted for modeling the TIS signal. For flank-contrasting methods, both TIS contexts and flanking sequences (96 nt in length per flank) are extracted for training signal and content sensors, respectively. A minimal distance between true and false TISs of 105 nt is used.

Common to all detection methods implemented in MetWAMer is utilization of a start-methionine signal-specific weight array matrix (WAM) that records position-specific base transition frequencies proximal to methionine codons in protein coding sequences. Here, WAMs characterize position-specific dinucleotide abundances; see the **Stratified training and testing **section below for a more detailed description. The train_MetWAM utility from the MetWAMer package can be used to develop such a WAM, given appropriate training data. The first in-frame methionine codon encountered, subsequent to a specified offset in the training sequence, is considered to be the true TIS for that sequence. Training of the methionine weight array matrix proceeds by tabulating dinucleotide frequencies from five positions upstream of the adenine through three positions downstream of the guanine residue of the pertinent methionine codon. Next, the system advances 105 bases in the training instance, to resume scanning for in-frame methionine codons, each of which will be classified as a false TIS; dinucleotide frequencies proximal to these false TISs are tabulated in the same manner as true TISs (see Figure 1). Following tabulation of dinucleotide frequencies at true and false TISs in training data, these are converted to relative frequencies, yielding the WAM, which enables calculation of the likelihood that a site in question is a true or false TIS.

#### Methionine log-likelihood ratios

The log-likelihood ratio (LLKR) approach to TIS prediction functions by scanning the ORF for in-frame ATG codons. (We use ATG to denote a methionine codon, as opposed to AUG, because MetWAMer scans for potential TISs in conceptually spliced genomic sequences.) A constraint is imposed on the protein length implied by any potential start-methionine such that if the ATG served as a true translation initiation site, the resulting protein must exceed 50 amino acid residues. Using the trained methionine-WAM, the method scores each such feasible site by calculating the likelihood that it is a true initiation site and taking the ratio of this value relative to the likelihood that it is not a true start site. The system identifies the methionine codon yielding the optimal value among such likelihood ratios, and provided the log of this ratio is non-negative, the LLKR routine returns it as the predicted start-methionine. The non-negativity constraint implements a classification threshold, imposed because we require the likelihood of the potential start site to favor its actually being a true TIS. If the system fails to identify any in-frame ATG codons, or the best-scoring site's score is negative-valued, then LLKR returns no prediction for the maximal ORF being surveyed.

#### Weighted methionine log-likelihood ratios

The weighted log-likelihood ratio approach (WLLKR) is identical to LLKR, but each in-frame ATG's log-likelihood ratio score is scaled as a function of the induced protein product's coverage of the maximal ORF. Precisely, coverage *x *is defined as the ratio of the length of the implied amino acid chain starting from the TIS under consideration over the length of the maximal ORF. For a true TIS, we expect the coverage value to be close to unity, as it would be unusual for a long, uninterrupted reading frame to be evolutionarily maintained in a genome, yet not be encoding an expressed, functional protein product. Empirically, we settled on weights calculated as *w*(*x*) = *x*^3 ^(other convex functions give commensurate results). The WLLKR routine optimizes over weighted log-likelihood ratios for all in-frame ATG codons, returning a predicted start-methionine if and only if the optimal such value is non-negative.

#### Multiplicative-based flank-contrasting with weighted methionine log-likelihood ratios

MetWAMer also implements an approach to start-methionine prediction that considers two descriptive features of potential TISs: weighted methionine log-likelihood ratio scores as used by the WLLKR routine (signal sensing) and the ratio of coding potential in a swath of sequence downstream from the site to that of a swath upstream of it, evaluated under a coding hypothesis (content sensing). Intuitively, we expect that the coding potential of the sequence downstream from a true site – which is, by definition, coding – would exceed that upstream of it – which is, by definition, non-coding – and that the ratio of the former to the latter should be greater in true sites as opposed to false. Coding probabilities of sequence swaths (96 nucleotides in length) are computed using a fifth-order *χ*^2^-interpolated Markov chain model [25,26] as implemented in the IMMpractical library [32]. The idea of integrating both content- and signal-based features into TIS prediction has been explored before [11,12,33], although the methodologies used here are distinct from previous studies.

For the multiplicative-based flank-contrasting with weighted methionine log-likelihood ratios (MFCWLLKR) method, the signal- and content-based scores, expressed in log space, are added. The system optimizes over these scores at viable, in-frame start-methionine sites, and if the best-scoring site's score is non-negative, it is returned by the routine as its TIS prediction.

#### Perceptron-based flank-contrasting with weighted methionine log-likelihood ratios

The perceptron-based flank-contrasting with weighted methionine log-likelihood ratios (PFCWLLKR) routine considers the same descriptive features as MFCWLLKR, but uses a perceptron as a multivariate utility function, as opposed to the multiplication operator. Perceptrons implement linear discriminants, and as such require linearly (or near-linearly) separable data sets to provide good classification performance (see, e.g., §4.1.7 of [34]). Intuitively, we expect that the two dimensions corresponding to the signal- and content-based features exhibit linear (or near-linear) separability: both weighted log-likelihood ratios of methionine sites and log-likelihood ratios of the coding potentials of downstream to upstream content swaths should be greater-valued in true start methionines as opposed to false, non-start ones. Linear and sigmoid units are used to implement perceptrons in the MetWAMer system; each of these neural elements can learn a continuous-valued function that can be thresholded to enable discrete, binary classification; excellent discussions of these methods can be found in §4.4.3 of [35] and §20.5 of [36]. Thus, linear and sigmoid units can be used to optimize over viable candidate start-methionine codons.

PFCWLLKR returns the best such potential TIS if and only if it is classified as being a true site by the perceptron. Although Stormo *et al*. used a perceptron to classify translation initiation sites in bacteria in a pioneering study [9], they considered an entirely distinct feature set.

#### Bayesian TIS prediction

Lastly, we also considered a Bayesian approach (BAYES) to predicting TIS sites. Each viable start-methionine in the maximal reading frame is considered under two separate models, one that the ATG is a true translation start codon and the other that it is not. The maximum *a posteriori *(MAP) hypothesis among this set of possibilities is computed, and if the site it denotes is represented as being a true TIS, BAYES returns this result as its TIS prediction. Otherwise, the method refrains from making any predictions. Calculation of the MAP hypothesis is formulated as follows. A prior distribution is derived for each maximal reading frame being surveyed: each in-frame ATG, under the model of its being a true initiation site, is given a prior probability proportional to the relative length of the peptide it induces compared with that of the maximal reading frame. Similarly, under the model of not being a TIS, each such site is assigned a prior probability proportional to the complement of its prior probability of being a true one. These values are normalized so as to collectively represent a valid probability mass function over all putative start-methionine sites, under both models. The likelihood of data is modeled using log-likelihood scores computed with the methionine-WAM.

### Data sets

Only gene annotations marked as curated in the current *Arabidopsis thaliana *annotation made available by TAIR (version 7, [37]) were used for developing methionine-weight array matrices. In TAIR, a curated status implies that these structures have been either manually inspected or are supported by full-length cDNA evidence. Training instances were further required to encode protein products at least 100 amino acid residues long, whose initial codon was ATG. For annotations satisfying these criteria, coding sequences were extracted from genomic templates using supplied reference coordinates. Because the TAIR annotation contains deliberate indel mutations in certain coding sequences with respect to genomic templates (see, e.g., gene models At1g03530.1  and At5g21105.1 ), and these modifications are not reflected in genomic reference coordinates, only parsed coding sequences having lengths divisible by three were retained for analysis. This overall process is implemented in the parse tigr codseqs utility from the MetWAMer package, which processes documents provided in the TIGR XML format [38].

These data were then post-processed to purge transposable elements and curtail redundancy. All coding sequences with significant matches (E-value < 10^-15^) to a sequence present in the TIGR plant repetitive element database [39], calculated using BLASTN [40], were eliminated. To limit redundancy in the remaining data, the BLASTClust utility [40] was used: sequence pairs having ≥ 80% nucleotide identity covering ≥ 80% of the longest sequence's length were clustered. Any sequence that clustered with one or more others was eliminated from the data set, i.e., we retained only one gene from each cluster. This resulted in 19,703 TIS-containing genes being retained for analysis.

A non-TIS-containing data set was compiled also, for testing the methods' abilities to not predict a TIS when none is present. The TIS-containing gene set was used as a starting point, from which we excluded single-exon genes. Of the remaining structures, the first coding exons (known to contain true TISs) were ablated from the conceptually spliced mRNAs; either 0, 1, or 2 bases were clipped from the 5'-terminus of these second exons in order to preserve the original reading frame. Next, sufficient flanking genomic sequences upstream of these exons were prepended, to facilitate the flank-contrasting methods – we remain neutral as to whether these are contributed entirely from the first ORF-disrupting intron of the gene, or if they might also include fragments from one or more upstream exons, 5'-UTR introns, or intergenic sequences. In total, 16,121 non-TIS-containing instances were retained for analysis.

### Stratified training and testing

In addition to homogeneous training, which does not address the possibility of characteristic features of potentially distinct biological classes of translation initiation sites, the calc_medoids utility of MetWAMer implements a method for developing stratified training data sets, which can be used to parameterize MetWAMer for cluster-specific TIS prediction behavior. The *k*-medoids algorithm, as implemented in the C Clustering Library [41], is used to calculate medoids (instances in each of the *k *clusters for which the distance to all other elements of the cluster are minimized), using a non-redundant set of translation initiation site sequences (five bases upstream of the ATG codon through three bases downstream). The Hamming distance is used to measure pairwise similarity of such instances.

MetWAMer implements a total of six possible methods for utilizing cluster-specific information during the prediction phase, when the true class of the sequence's TIS is unknown beforehand: three distinct measures of a site's "closeness" to those in a given cluster are defined, and each measure can be used either by selecting the best parameter set for every site encountered during scanning (modulating) or by choosing the best set on the basis of the first in-frame ATG encountered, and committing to the exclusive use of it for scoring any remaining putative TISs in the reading frame (static). Thus, these combinations comprise a collection of parameter set indexing strategies, which allow for lookup of those partition-specific parameters most appropriate for scoring a site.

The first measure considered is the Hamming distance, which lends itself to an indexing strategy in which, for a putative TIS, its distance is computed relative to the *k *medoids identified in the clustering step; cluster-specific parameters corresponding to the medoid whose Hamming distance is minimal to it, are used to score. The PWM-based indexing method utilizes cluster-specific position weight matrices for measuring the site's similarity to known clusters, and the parameter set whose representative PWM renders the putative TIS most likely is used for scoring. Specifically, a PWM characterizes position-specific mononucleotide distributions at genetic elements such as promoter sites, splice sites, or translation initiation sites [42]. Here, the likelihood of a potential TIS as having been generated by the (trained) PWM is given by

$$LK_{pwm}=\prod_{i=-5...+5}f_{B_{i}},$$

where *i *indexes each position in the site (the adenine residue of the ATG codon is assigned position 0), and $f_{B_{i}}$ is the relative frequency of base *B*_*i *_∈ {*A*, *C*, *G*, *T*} in position *i *of the aligned training sequences. Finally, a WAM-based indexing method is implemented, which is analogous to the PWM-based strategy, though (first-order) weight array matrices are used for computing likelihoods, rather than PWMs. For a potential TIS site, the likelihood of its having been generated by the WAM is computed by MetWAMer as

$$LK_{wam}=\prod_{i=-5...+4}f_{D_{i,i+1}},$$

where *i *indexes each position in the site, and $f_{D_{i,i+1}}\in \{AA,AC,AG,...,TT\}$ is the relative frequency of the observed dinucleotide *D*_*i*,*i*+1 _occurring at position *i *in aligned training data.

### Test design

**Figure 2:**
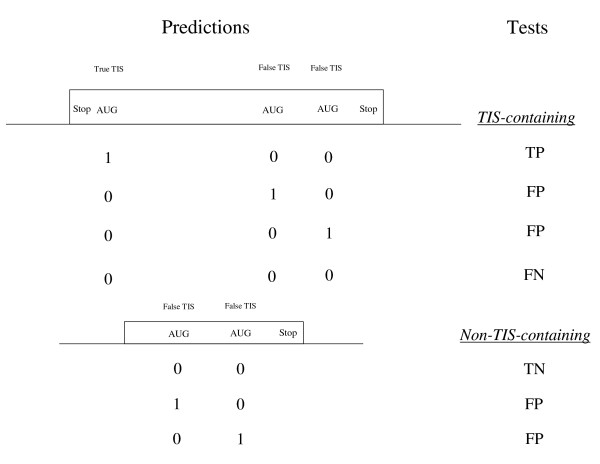
**TIS detection competency tests**. Shown are two distinct testing scenarios for TIS identification competency in maximal, TIS-containing reading frames and in reading frames lacking a true TIS. In TIS-containing tests, three outcomes are possible: the system predicts the true TIS as the TIS for the gene (TP), it predicts a false TIS as the gene's TIS (FP), or it fails to predict any TIS for the gene (FN). In the non-TIS-containing scenario, the system either (correctly) refuses to predict a TIS for the gene (TN) or mislabels some in-frame ATG as a TIS (FP).

A five-fold cross-validation strategy was used to assess the methods on the task of translation initiation site detection competency. Because the TIS-containing instances consist only of known coding sequences from gene structures, and in practice MetWAMer scans for potential TISs across a maximal ORF, we extended the coding sequences at their 5'-termini to achieve a maximal, non-stop reading frame, thereby presenting the system with the challenge of disambiguating spurious (in-frame) methionine codons in the extended reading frame from true start codons – we make no considerations as to whether these extended sequences are derived from 5'-UTRs, introns in 5'-UTRs, or intergenic sequences. Methionine-WAMs and Markov chains were trained on each cross-validation replicate and then used to train a sigmoidal perceptron, using a learning rate of 1 × 10^-5^. Sigmoid units outperformed linear units in all experiments we conducted (data not show), so we do not consider the latter further. As a baseline for comparison of the implemented models, we consider also the 1^*st*^-ATG method, which predicts the first in-frame ATG it encounters in the maximized reading frame as a TIS. Tests using non-TIS-containing instances were conducted similarly, though reading frames were not maximally extended at their 5'-terminus. The testing procedure is shown pictorially in Figure 2.

To assess the performance of MetWAMer relative to prior art in translation initiation prediction, we compared our system with the NetStart [10], TIS Miner [43], TISHunter [17] and ATGpr [12] programs.

## Results

### Computational TIS identification in TIS-containing ORFs

Table 1 summarizes TIS prediction accuracy in TIS-containing ORFs. Given the knowledge that the transcript under consideration is 5'-complete, the simple strategy of predicting the leftmost ATG to be the TIS ("1^*st*^-ATG") is seen to give the best performance by far. The 94% sensitivity and specificity merely reflects the proportion of transcripts not subject to leaky scanning. All other methods incorporate uncertainty about 5'-completeness and specifically allow for the possibility of observing a non-TIS-containing transcript fragment (prediction of which for this test set would always result in a false negative instance). Restricting attention to the method-specific results obtained under homogeneous parameter usage, it can be seen that MFCWLLKR has better sensitivity than the remaining methods, with PFCWLLKR exhibiting comparable levels. PFCWLLKR dominates the remaining models in terms of specificity. WLLKR is the third-most successful method at identifying true TISs, though it suffers from a relatively high rate of false negative predictions. The BAYES routine makes fewer true positive predictions than WLLKR, and more false positive and false negative identifications.

**Table 1:** Method performances on TIS-containing data.

Parametrization	Method	TP	FP	FN	Sn	Sp
	1^*st*^-ATG	18,553	1,150	0	0.9416	0.9416
	TISHunter	17,789	1,914	0	0.9029	0.9029
	ATGpr	17,160	2,543	0	0.8709	0.8709
	TIS Miner	15,521	3,650	532	0.7877	0.8096
	NetStart	5,123	14,527	53	0.2600	0.2607
						
homogeneous	LLKR	9,268	9,318	1,117	0.4704	0.4987
	WLLKR	12,511	4,486	2,706	0.6350	0.7361
	MFCWLLKR	15,167	4,535	1	0.7698	0.7698
	PFCWLLKR	14,692	4,191	820	0.7457	0.7781
	BAYES	10,121	6,482	3,100	0.5137	0.6096
						
cluster-specific	LLKR	11,964	6,946	793	0.6072	0.6327
	WLLKR	14,931	3,085	1,687	0.7578	0.8288
	MFCWLLKR	16,576	3,127	0	0.8413	0.8413
	PFCWLLKR	16,209	2,834	660	0.8227	0.8512
	BAYES	12,399	4,988	2,316	0.6293	0.7131
						
random split	LLKR	9,191	9,402	1,110	0.4665	0.4943
	WLLKR	12,491	4,507	2,705	0.6340	0.7349
	MFCWLLKR	15,183	4,519	1	0.7706	0.7706
	PFCWLLKR	14,648	4,198	857	0.7434	0.7772
	BAYES	10,084	6,509	3,110	0.5118	0.6077

19,703 TIS-containing instances were used in three separate five-fold cross-validation experiments. Results are shown from applying a non-stratified parameter set (homogeneous), *a priori*-known cluster-specific parameter sets for *k *= 3 (cluster-specific), and group-specific parameter sets for a random three-way split of the data (random split). *TP *represents the number of instances for which the method correctly identified a TIS; *FP *for which a prediction was made, though incorrect; and *FN *for which no prediction was made, but should have been (see Figure 2). $Sn=\frac{TP}{TP+FP+FN}$, and $Sp=\frac{TP}{TP+FP}$.

Cluster-specific parameter results were produced by first stratifying the data with respect to the clusters identified by *k*-medoids, for *k *= 3, conducting five-fold cross-validation analyses independently for each cluster, and averaging the results. Thus, we explicitly leveraged information concerning the true cluster to which a test sequence's TIS belongs. All methods increased markedly in TIS prediction performance. To demonstrate that this observation is not simply an artifact due to potentially over-fitting the models to smaller training set sizes, we randomly split the data into three separate partitions and repeated the analysis. The random split results are essentially indistinguishable from those obtained using homogeneous deployment, and thus we may conclude that the performance gains from cluster-specific parameter training reflect non-random effects.

### Computational TIS identification in transcripts undergoing leaky scanning

1,150 instances from the TIS-containing data set are known to contain in-frame ATGs upstream from the true TIS. Table 2 provides TIS prediction statistics derived exclusively from these cases. By definition, 1^*st*^-ATG is a complete failure in this scenario. PFCWLLKR has greater sensitivity than all other methods under all parameter deployment strategies with the exception of cluster-specific, in which MFCWLLKR bests it by roughly 0.35%. WLLKR strictly dominates all methods in terms of specificity, outperforming the second-best method, PFCWLLKR, by under four percent for any parametrization strategy.

**Table 2:** Distinguishing in-frame, upstream ATG sites from true TISs

Parametrization	Method	TP	FP	FN	Sn	Sp
	1^*st*^-ATG	0	1,150	0	0.0000	0.0000
	TISHunter	69	1,081	0	0.0600	0.0600
	ATGpr	97	1,053	0	0.0843	0.0843
	TIS Miner	216	832	102	0.1878	0.2061
	NetStart	216	930	4	0.1878	0.1885
						
homogeneous	LLKR	437	671	42	0.3800	0.3944
	WLLKR	531	467	152	0.4617	0.5321
	MFCWLLKR	525	625	0	0.4565	0.4565
	PFCWLLKR	542	551	57	0.4713	0.4959
	BAYES	442	511	197	0.3843	0.4638
						
cluster-specific	LLKR	552	550	48	0.4800	0.5009
	WLLKR	663	380	107	0.5765	0.6357
	MFCWLLKR	683	467	0	0.5939	0.5939
	PFCWLLKR	679	419	52	0.5904	0.6184
	BAYES	567	406	177	0.4930	0.5827
						
random split	LLKR	434	672	44	0.3774	0.3924
	WLLKR	522	468	160	0.4539	0.5273
	MFCWLLKR	530	620	0	0.4609	0.4609
	PFCWLLKR	541	551	58	0.4704	0.4954
	BAYES	439	512	199	0.3817	0.4616

TIS identification statistics are reported exclusively for the 1,150 maximal reading frames containing in-frame ATG sites upstream from the true TIS, under three distinct parameter utilization approaches: homogeneous, *a priori*-known cluster-specific with *k *= 3, and three-fold random split. *TP *represents the number of instances for which the method correctly identified a TIS; *FP *for which a prediction was made, though incorrect; and *FN *for which no prediction was made, but should have been (see Figure 2). $Sn=\frac{TP}{TP+FP+FN}$, and $Sp=\frac{TP}{TP+FP}$.

PFCWLLKR should be more prone to false positive prediction on these sequences because the upstream ATGs would typically have better coding potential contrast than the true TIS.

### Method performace on non-TIS-containing transcript fragments

Table 3 provides TIS prediction performance statistics in non-TIS-containing instances. 1^*st*^-ATG performs worse than all other methods, under every deployment approach. Under homogeneous parameter deployment, WLLKR dominates the remaining methods, with BAYES being second-best, and PFCWLLKR third-best, with sensitivities varying in a range of less than three percent. Again, it is observed that cluster-specific parameter usage leads to considerable performance gains, whereas random splits produce results essentially indistinguishable from the homogeneous-based results.

**Table 3:** Method performances on non-TIS-containing data

Parametrization	Method	TN	FP	Sn
	1^*st*^-ATG	688	15,433	0.0427
	TISHunter	0	16,121	0.0000
	ATGpr	0	16,121	0.0000
	TIS Miner	3,142	12,979	0.1949
	NetStart	575	15,546	0.0357
				
homogeneous	LLKR	5,179	10,942	0.3213
	WLLKR	7,260	8,861	0.4503
	MFCWLLKR	1,688	14,433	0.1047
	PFCWLLKR	6,785	9,336	0.4209
	BAYES	6,813	9,308	0.4226
				
cluster-specific	LLKR	6,385	9,736	0.3961
	WLLKR	8,080	8,041	0.5012
	MFCWLLKR	1,995	14,126	0.1238
	PFCWLLKR	8,685	7,436	0.5387
	BAYES	8,057	8,064	0.4998
				
random split	LLKR	5,155	10,966	0.3198
	WLLKR	7,176	8,945	0.4451
	MFCWLLKR	1,687	14,434	0.1046
	PFCWLLKR	6,748	9,373	0.4186
	BAYES	6,824	9,297	0.4233

16,121 non-TIS-containing instances were used in three separate five-fold cross-validation experiments. Results are shown from applying a non-stratified parameter set (homogeneous), *a priori*-known cluster-specific parameter sets for *k *= 3 (cluster-specific), and group-specific parameter sets for a random three-way split of the data (random split). *TN *represents the number of instances for which the method (correctly) refused to predict a TIS, and *FP *denotes the number for which some prediction was made, though always incorrect (see Figure 2). $Sn=\frac{TN}{TN+FP}$.

### Comparison with other TIS prediction tools

Based on results shown in Tables 1, 2, 3, we identify PFCWLLKR as the superior method currently implemented in MetWAMer, and therefore used it as a benchmark for comparison with other TIS prediction tools. Specifically, we consider PFCWLLKR used under the homogeneous parameter deployment approach. We compare this method with the NetStart [10], TIS Miner [43], TISHunter [17] and ATGpr [12] programs. Because NetStart is a TIS classifier, and not a TIS prediction system, we interpreted its results as follows. For all potential TISs scored by the program, we ranked each instance on the basis of its score. If the best-scoring instance was classified as a true TIS (marked "Yes"), it was selected as the program's single TIS prediction; else, we interpreted the result as the system's decision to make no TIS prediction at all. We used the web interface to the program available at  and used its *Arabidopsis*-specific parameters. The TIS Miner program, available at  was used with default paramters, with the number of predictions set to 1. We used a classification threshold of 0.5 for this program, such that if the TIS prediction it returned was at least 0.5, it was selected as the system's prediction, while if not, this was interpreted as its decision not to return a TIS prediction. This threshold setting performed best over a range of values tried (data not shown). Finally, the TISHunter and ATGpr programs, available at  and , respectively, were used with default settings. All raw output generated by these tools on our test data is available as supplementary information at [29].

As depicted in Table 1, PFCWLLKR handily outperforms the NetStart system, though it is bested by the TIS Miner (albeit by a slight margin), TISHunter and ATGpr programs on these TIS-containing instances. In no case are the competing programs able to outperform 1^*st*^-ATG. Table 2 demonstrates that PFCWLLKR is considerably better than the competing methods at identifying a true TIS when an in-frame site occurs upstream from it, however. Finally, Table 3 shows that PFCWLLKR is far better at declining to predict a TIS when none are present than any of the four competing programs.

### Performance gains by parameter set indexing

Based on the results shown in Tables 1, 2 and 3, we decided to focus on the PFCWLLKR method in the following. Indeed, although we assessed all the methods in the experiments described below, PFCWLLKR was superior in all cases (data not shown).

As shown in Tables 1 and 3, all parametric methods exhibited an increase in successful TIS identification when using stratified parameter sets, suggesting that considerable improvements in statistically-based models for TIS prediction can be achieved by taking the appropriately defined class of each potential start-methionine into account. This motivated the development of a lookup method for indexing appropriate parameter sets when the class of a test sequence's true TIS is not known beforehand. Table 4 provides results obtained using PFCWLLKR on TIS-containing data, under the six parameter indexing schemes described in the **Implementation **subsection **Stratified training and testing**. For any given value of *k *∈ {3, 5, 10}, static WAM-based indexing performs best overall. Additionally, increasing values of *k *resulted in increased performance on these data. All indexing approaches improved under static parameter lookup relative to modulating, for all deployment strategies. This can be explained given the observation that in-frame ATGs upstream from true TISs are relatively rare, e.g., they occur with a frequency of roughly 1,150/19,703 ≈6% in *Arabidopsis*, and thus, provided the similarity measure used can recover the site's corresponding class with good fidelity, performance should closely approximate that obtained under a *priori*-known cluster-specific parameter usage.

**Table 4:** Effect of parameter set indexing strategy on PFCWLLKR performance using TIS-containing data

*k*	Indexing strategy		TP	FP	FN	Sn	Sp
3	modulating	edit	14,395	4,944	364	0.7306	0.7444
		PWM	14,270	5,020	413	0.7243	0.7398
		WAM	14,388	4,949	366	0.7302	0.7441
							
	static	edit	15,895	3,157	651	0.8067	0.8343
		PWM	15,757	3,226	720	0.7997	0.8301
		WAM	15,916	3,158	629	0.8078	0.8344

5	modulating	edit	13,753	5,540	410	0.6980	0.7128
		PWM	13,856	5,501	346	0.7032	0.7158
		WAM	14,208	5,267	228	0.7211	0.7296
							
	static	edit	15,908	2,781	1,014	0.8074	0.8512
		PWM	16,080	2,704	919	0.8161	0.8560
		WAM	16,454	2,634	615	0.8351	0.8620

10	modulating	edit	12,849	6,364	490	0.6521	0.6688
		PWM	13,861	5,647	195	0.7035	0.7105
		WAM	14,169	5,422	112	0.7191	0.7232
							
	static	edit	15,729	2,441	1,533	0.7983	0.8657
		PWM	16,755	2,135	813	0.8504	0.8870
		WAM	17,156	2,013	534	0.8707	0.8950

19,703 TIS-containing instances were used in five-fold cross-validation experiments, in which parameter sets were selected for putative TIS evaluation according to best cluster fit established by either the Hamming distance relative to cached medoids (edit), position weight matrix scores (PWM), or weight array matrix scores (WAM). Parameter indexing was tested under both modulating (cluster assignment for each site separately) and static (cluster assignment based on the leftmost ATG) approaches. *k *denotes the number of clusters considered. *TP *represents the number of instances for which the method correctly identified a TIS; *FP *for which a prediction was made, though incorrect; and *FN *for which no prediction was made, but should have been (see Figure 2). $Sn=\frac{TP}{TP+FP+FN}$, and $Sp=\frac{TP}{TP+FP}$.

Table 5 presents results analogous to Table 4, for non-TIS-containing data. Again, static parameter lookup yielded results superior to those obtained under the modulating approach. Increases in *k *typically resulted in a greater number of false positive predictions being made, resulting in progressively lower performance.

**Table 5:** Effect of parameter set indexing strategy on PFCWLLKR performance using non-TIS-containing data

*k*	Indexing strategy		TN	FP	Sn
3	modulating	edit	5,074	11,047	0.3147
		PWM	5,134	10,987	0.3185
		WAM	5,069	11,052	0.3144
					
	static	edit	6,170	9,951	0.3827
		PWM	6,279	9,842	0.3895
		WAM	6,119	10,002	0.3796

5	modulating	edit	4,537	11,584	0.2814
		PWM	4,484	11,637	0.2781
		WAM	4,262	11,859	0.2644
					
	static	edit	5,993	10,128	0.3718
		PWM	6,065	10,056	0.3762
		WAM	5,679	10,442	0.3523

10	modulating	edit	4,190	11,931	0.2599
		PWM	3,708	12,413	0.2300
		WAM	3,533	12,588	0.2192
					
	static	edit	6,345	9,776	0.3936
		PWM	5,537	10,584	0.3435
		WAM	5,199	10,922	0.3225

16,121 non-TIS-containing instances were used in five-fold cross-validation experiments, in which parameter sets were selected for putative TIS evaluation according to best cluster fit established by either the Hamming distance relative to cached medoids (edit), position weight matrix scores (PWM), or weight array matrix scores (WAM). Parameter indexing was tested under both modulating (cluster assignment for each site separately) and static (cluster assignment based on the leftmost ATG) approaches. *k *denotes the number of clusters considered. *TN *represents the number of instances for which the method (correctly) refused to predict a TIS, and *FP *the number for which some prediction was made, though always incorrect (see Figure 2). $Sn=\frac{TN}{TN+FP}$.

### MetWAMer as a TIS classifier

**Figure 3:**
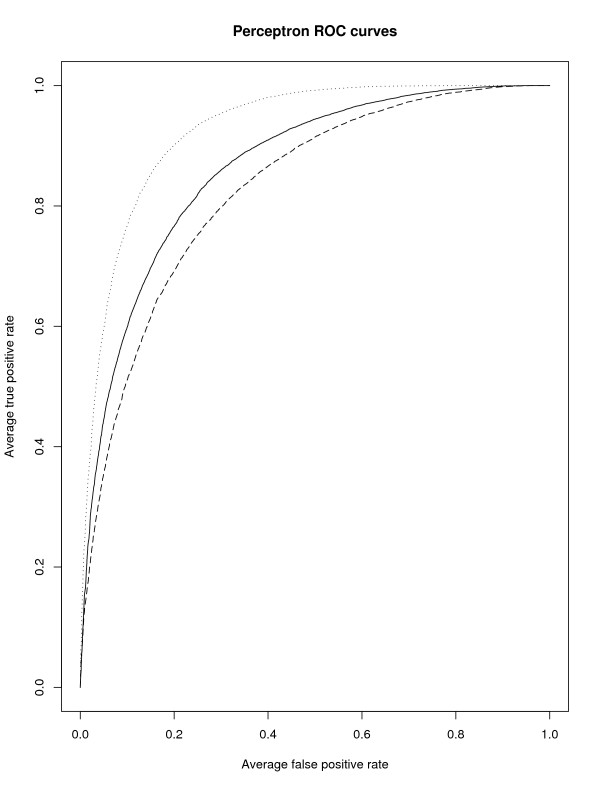
**Receiver operating characteristic curves for the perceptron element of PFCWLLKR**. The classifier was assessed on the task of distinguishing ATG codons as true or false TISs, under distinct parameter deployment strategies: the dotted curve denotes perceptron performance obtained under *a priori*-known cluster-specific parameter usage, the solid curve that from homogeneous parameter deployment, and the dashed curve from WAM-based parameter set indexing. A true positive is defined as a true TIS labeled as such, whereas a false positive denotes a false TIS labeled by the classifier as true. These plots were generated using the ROCR package [62].

Although MetWAMer is not a TIS classifier *per se*, each TIS prediction method utilizes some form of discriminant technique with which to evaluate whether the best-scoring in-frame ATG (a putative TIS) is a true or false site. Figure 3 shows receiver operating characteristic (ROC) curves for the sigmoidal perceptron element of PFCWLLKR, which was assayed on the task of labeling ATG codons as true or false TISs under distinct parameter deployment strategies. Five-fold cross-validation was used to classify 34,229 instances, 19,703 of which were known TISs from the TIS-containing gene set and 14,526 of which were the first in-frame ATG codons (false TISs) from the non-TIS-containing set (the negative instances number fewer than the 16,121 instances used in Table 3 because 1,595 of the truncated, multiple-exon gene structures lacked any in-frame ATG). The ROC plots demonstrate that utilization of a *priori*-known cluster-specific parameter sets yields a classifier superior to that obtained using a single, homogeneous set. However, WAM-based indexing yielded a classifier worse than both the others. This seems due to the comparatively worse performance of the parameter set lookup strategies in general at rejecting false TISs (e.g., compare PFCWLLKR performance results in Table 3 with those in Table 5).

### Biological interpretation of TIS classes

**Figure 4:**
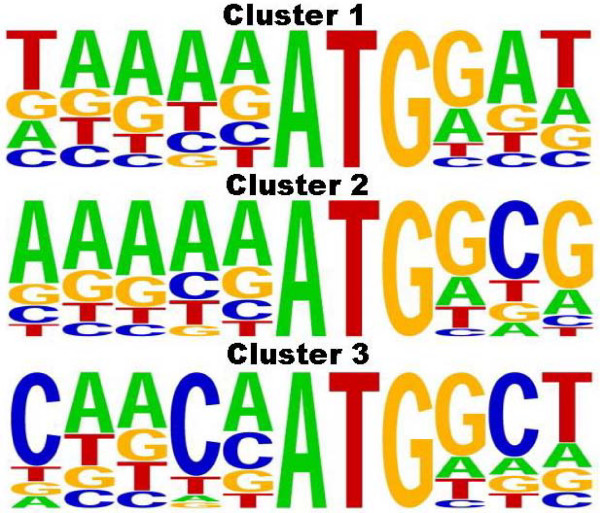
**Cluster-specific TIS mononucleotide distributions**. Sequence logo plots [63], depicting site-specific nucleotide abundances, were generated for TIS sequences obtained from clusters 1 through 3 using the WebLogo utility [64]. The medoids computed by the *k*-medoids algorithm for clusters 1 through 3 are TAAAAATGGAT, AAAAAATGGCG, and CAACAATGGCT, respectively.

Given the improved performance of the methods under the *a priori*-known cluster-specific parameter deployment strategy, we wondered if any underlying biological basis for the grouping obtained by *k*-medoids, for *k *= 3, may exist. Clustering was performed using a non-redundant set of TISs, and the cluster-specific consensus sequences derived from position-specific mononucleotide distributions perfectly recovered each cluster's associated medoid (see Figure 4). While these consensus sequences were fairly weak – as evidenced by the high degree of entropy at each position in the TIS alignment – the observation nevertheless indicates that the clustering algorithm's results are meaningful, and also suggests the possibility of at least three distinct groups of TISs in *Arabidopsis*. The possibility that these could correspond to distinct gene classes was explored using a non-parametric statistical test on ontological annotations, in which the significance of cluster-specific distributions of GOslim (specifically, cellular component) terms [44] was determined by sampling same-size sets from the full population of terms. We labeled a cluster as being over- or underrepresented with respect to a particular GOslim term if its frequency in the class was in the top or bottom five values in comparison with 99 randomly-sampled sets, respectively. Clusters 1 through 3 contained 6,298; 5,039; and 3,019 instances having associated GOslim terms, respectively, with the overall population containing 14,356 terms. Our results, presented in Table 6, suggest that cluster 1 is largely depleted of plastid and ribosomal genes, while cluster 2 is enriched for these; cluster 3 is enriched for plastid and cytosolic genes. However, these observations should perhaps be deemed inconclusive, as many genes in our data set do not yet have associated GOslim terms, and for those that did, such annotations should typically be considered tenuous at present.

**Table 6:** Cluster-specific over- and underrepresentation of GOslim terms

GOslim term	High	Low	Normal
cell wall			1,2,3
chloroplast	3	1	2
cytosol	3	1	2
ER			1,2,3
extracellular		3	1,2
Golgi apparatus			1,2,3
mitochondria	2	1	3
nucleus	1	2,3	
other cellular components			1,2,3
other cytoplasmic components	2		1,3
other intracellular components			1,2,3
other membranes			1,2,3
plasma membrane			1,2,3
plastid	2,3	1	
ribosome	2	1	3
unknown cellular components	1	2,3	

*Arabidopsis *transcripts were clustered into three sequence clusters based on TIS similarity. Within these clusters, the numbers of gene models with associated GOslim terms are, respectively, 6,298, 5,039, and 3,019. Clusters denoted as being "High" for a specific term were determined to be enriched in genes labeled as such, relative to the full population of terms, by a randomization test. Those labeled "Low" were found to be relatively impoverished in genes labeled with the associated term, and "Normal" as being neither significantly over- nor underrepresented.

## Discussion

Our results on the TIS-containing data set suggest that, compared with the methods implemented in MetWAMer, a policy of labeling the first ATG as TIS in a maximal ORF wil achieve quite good (though imperfect) results. However, in practice we cannot always assert whether a maximal ORF has sufficient 5'-coverage so as to include the gene's true TIS, or whether a spurious in-frame ATG occurs upstream from it. In such cases, the 1^*st*^-ATG strategy fails, as it does in cases of leaky scanning, thus sustaining the importance of further development of statistical TIS prediction methodologies that capture the sequence features recognized by the ribosome in translation initiation. In this work, we present a number of distinct models for TIS prediction, the most successful of which mixes content- and signal-based features of putative TISs using a perceptron (PFCWLLKR). Furthermore, we demonstrate that, in the model plant *Arabidopsis*, TIS prediction can be enhanced by integration of class-specific parameter sets, regardless of the prediction method utilized.

We attribute the well-balanced performance of PFCWLLKR to the biological plausibility of the features provided to it as inputs. As a signal-based feature, weighted log-likelihood ratios considerably improve the specificity of TIS prediction (e.g., contrast WLLKR and LLKR in Tables 1 and 2, likely because our weighting function, *w*(*x*) = *x*^3 ^for induced protein length to maximal ORF coverage *x*, appears to empirically approximate the epistemology of eukaryotic translation initiation fairly well: according to the (leaky) ribosomal scanning hypothesis [3], one would expect that more upstream AUG sites – especially those occurring in a favorable signaling context – in a maximal reading frame would be more likely to function as *bona fide *translation initiation sites. Also, it is unusual for a long, uninterrupted reading frame to be maintained, yet not expressed as part of a functional protein product. Our weighting scheme has been explicitly designed to reflect these biologically-informed biases.

During the post-scanning phase of translation initiation, the small ribosomal subunit stalls at a TIS to recruit the large subunit, thereby forming the 80S ribosomal particle. The scanning process, as conducted by the small ribosomal subunit in concert with various eukaryotic initiation factors, does not appear to take more global nucleotide compositional features of the mRNA molecule into account, notwithstanding the possibility of secondary structures causing steric interference with scanning itself. That we might utilize contrast in coding potential of sequences flanking a TIS for modeling purposes is a consequence of the fact that sequences upstream of a TIS are non-coding, and those downstream, coding, though this plays no known role in the recognition of TISs *in vivo*. The use of Markov chains in a classification setting was shown to distinguish exons from introns with good accuracy in plant systems [32], and our expectation that these content-sensing tools could be gainfully transferred to the TIS prediction domain was born out by the performance results shown. Similar inclusion of coding potential contrast has also been employed to increase splice site prediction accuracy [31,45].

Our data set was developed from gene models flagged as curated in the current *Arabidopsis *annotations, though it should not be overlooked that potential errors in these structures might have distorted our results. Manual inspection of several genes whose TISs were predicted incorrectly by the PFCWLLKR routine indicate possible problems with existing annotations. For example, in gene model At4g34080.1 , our system predicted the TIS as that from the TAIR version 6 gene annotation, rather than that of version 7, which occurs downstream. Similarly, we predict the version 6 TIS of gene model At5g35580.1  as correct, rather than the revised TIS from the version 7 model. Partial protein sequencing using Edman degradation could potentially resolve such ambiguities in the annotations (e.g., [7]), as might consideration of homologous proteins with matching N-termini whose translation initiations sites had previously been determined; such efforts are beyond the scope of this work, however.

Although we were unable to achieve the performance levels of *a priori*-known cluster-specific parameter deployment with our parameter set indexing schemes, stratified parameter deployment can nevertheless be used effectively in practice, pending certain characteristics of the test data: if these are expected to be moderately enriched for 5'-complete sequences, then static WAM-based indexing should recover a larger fraction of true TISs than would homogeneous deployment. However, if complete 5'-coverage is expected to be quite sparse, homogeneous parameter deployment should be utilized instead. This affords a complete prescription of how to most effectively identify TISs in transcript data: 1^*st*^-ATG would be the best method for use in data sets with a high degree of 5'-completeness, static WAM-based PFCWLLKR in moderately enriched data sets, and homogeneous deployment of PFCWLLKR in data sets likely to contain few 5'-complete sequences.

We have replicated our experiments using a data set based on the most recent GenBank annotations for the nematode *Caenorhabditis elegans *(dated 16 February 2006), the results of which are similar to those presented here for *Arabidopsis *(available as supplementary material at [29]), suggesting that our method is not specific to plant taxa, and can be used for eukaryotic TIS prediction in general. Also available as supplementary material are homogeneous parameter deployment-based results for a small set of TIS-containing human genes culled from the Consensus CDS project [46]; these results imply that the system can be utilized for vertebrate taxa, as well.

As a demonstration of MetWAMer's applicability for post-processing gene structures predicted by separate tools, we refined maize gene annotations generated by the GeneSeqer spliced alignment program [47]. 11,742 full length maize cDNA sequences were obtained from the Maize Full Length cDNA project [48] and aligned via GeneSeqer to a set of 17,163 BAC sequences downloaded from PlantGDB [49]. These results were post-processed with MetWAMer's PFCWLLKR routine under homogeneous parameter deployment, using parameters trained with *Arabidopsis *data. We considered only predicted protein sequences such that at least one full length cDNA supporting its annotation exhibited an overall GeneSeqer alignment score of at least 0.9 and the predicted TIS occurred in or upstream from the first exon identified by spliced alignment. The resulting set of 6,926 proteins was aligned against a collection of 36,338 annotated sorghum proteins downloaded from the Phytozome project [50] using BLASTP. BLASTP output was inspected using the MuSeqBox program [51] in order to select only those inferred maize proteins of at least 150 amino acids in length whose best hit in the sorghum data, also at least 150 amino acids long, shared high-scoring segment pairs (HSPs) of at least 20% identity apiece such that the sum of these non-overlapping HSPs was not less than 90% of the length of either sequence. Furthermore, at most five amino acids at both the N-and C-termini, for both sequences, were allowed to be disjoint from an HSP. These 2,315 proteins were then made non-redundant using BLASTClust with default settings. In summary, the resultant set of 1,665 maize proteins on 1,463 distinct BACs identifed by GeneSeqer in concert with MetWAMer represents a reliable collection of high-quality, non-redundant full length maize proteins that could not have been identified by GeneSeqer alone, thereby demonstrating the practical utility of this approach to modern genome annotation projects. Our results are available as supplementary data at [29].

We compared annotation results of our pipeline with those achieved by a current state-of-the-art *ab initio *gene prediction tool, AUGUSTUS [52]. The BAC sequences containing our annotated maize genes were fed to the program and processed using its maize-specific parameters. We note that a fair comparison between the two approaches is basically impossible, since the search space probed by pure *ab initio *gene finders is quite distinct from that explored by spliced alignment annotation systems such as GeneSeqer+MetWAMer, so we disregard false positive predictions generated by AUGUSTUS. In summary, of the 1,665 maize proteins we identified, AUGUSTUS correctly predicted 1,232 (≈74%) TISs and 581 (≈35%) complete gene structures. These results underscore the necessity that a complete and robust gene annotation pipeline should integrate evidence from multiple data sources, gene prediction software and even manual gene curation results, as is achieved by various higher-order systems including AUGUSTUS+ [53], the Ensembl pipeline [54], EuGéne [55], and JigSaw [56,57]. Our efforts to integrate a variety of retrained, state-of-the-art gene finding tools using such systems in the context of various plant genomes will be presented in a forthcoming report.

## Conclusion

MetWAMer performance results, particularly for PFCWLLKR, suggest that the method can be used with good success for the task of annotating TISs in eukaryotes. However, our data are not precisely comparable with those provided by a number of previous studies, e.g., [10-13,15,19,33], just as results between those papers are essentially incomparable, as well. This is due to differing experimental designs (some studies focus on the number of ATG codons correctly classified as true or false TISs, and others on the number of genes for which the TIS was correctly identified) and different data sets (some studies used human genes, some cyanobacterial, etc., and these corpora were often of very different sizes).

Comparing these published methods with our own, using our data and experimental design, was often not practical: the availability of software implementing methods developed for eukaryotic TIS prediction *per se *is very limited at present. Among the papers addressing intrinsic TIS detection methods, only the ATGpr [12], StartScan [33], DIANA-TIS [11], TISHunter [17], NetStart [10], and TIS Miner [43] systems are described as "available" software. We were only able to utilize the NetStart, TIS Miner, TISHunter and ATGpr systems to compare against our software system, though we note that it is impossible to re-train any of these programs. StartScan is available via a web interface (currently trained only for human), but an important distinction from our tool is that StartScan is for TIS recognition in genomic sequences, a much different task than that addressed by MetWAMer. Although not mentioned in its reference paper, [11], we were able to locate a web interface to the DIANA-TIS system at the author's web page . However, documentation for the interface is unavailable, and most prohibitive is that it only allows a pictorial representation of its predictions, which is unrealistic for processing data sets of the scale used in this study. GeneHackerTL is mentioned in [19], but it is not described as being publicly available, nor were we able to locate it in any web-accessible forum.

The paucity of freely available, functioning programs for TIS prediction comprises an important gap in the software infrastructure for computational biology. Our MetWAMer package represents a well-documented, extensible, and open source software system that can be modified for differing applications and extended with existing and novel TIS prediction methods to support further research in this area; this is, to our knowledge, the first such contribution made to the eukaryotic TIS prediction community at-large. There are certain limitations to the existing scope of MetWAMer, however, which may present opportunities for future work. We have explicitly ignored the possibility of non-AUG start codons, although these are known to occur in various eukaryotic organisms [7,8]. Also, the system does not explicitly integrate extrinsic information, such as homologous proteins, which is reportedly successful [58]; however, due to evolutionary forces operating on homologous genes, it is possible that translation initiation sites differ, and the use of such information for prediction could be misleading. We have explicitly ignored the possibility of translation initiation proceeding by a re-initiation mechanism, whereby a short ORF upstream of the more significant ORF is translated, and the ribosome resumes translation at a downstream AUG [59]. For MetWAMer, however, this is not a potentially obfuscating phenomena: because the system scans for TISs in a maximal reading frame, there is no possibility to predict a start codon upstream of the significant ORF that is succeeded by a stop codon a short distance thereafter. Another open problem is the prediction of alternative TISs in various gene structures [60].

The ability to train TIS models in a species-specific manner is an important strength of MetWAMer, because differences in translation initiation processes among distinct taxa are known to occur [61]. To the extent that cross-specific TISs are representative of some target species, these could in principle be used as a proxy if species-specific data are not available; the performance of our system in such a scenario will be reported in a forthcoming study in which we refine gene structure annotations of a variety of cereal crop genomes. Results presented here also indicate that improvements in TIS prediction accuracy are possible when taking the class of potential start-methionines into account. Our software readily accommodates these needs, and can be integrated into other gene annotation programs and/or pipelines with straightforward modifications.

## Availability and requirements

• Project name: MetWAMer

• Project home page: 

• Operating system(s): Platform independent

• Programming language: C

• Other requirements: libxml2 version 2-6-23 or later , and IMMpractical version 1.0 or later  – see the MetWAMer manual page for details.

• License: ISC license

• Restrictions to use by non-academics: None

## Authors' contributions

VB suggested the project and advised on the models, experimental design, and manuscript. MES co-designed the models with VB, implemented the software, conducted experiments, and wrote the manuscript.

## Supplementary Material

Additional file 1 **MetWAMer.v1.3.** Source code for the MetWAMer package. This version was used to generate data reported in this study. Click here for file
